# Balanced modulation of neuromuscular synaptic transmission via M1 and M2 muscarinic receptors during inhibition of cholinesterases

**DOI:** 10.1038/s41598-022-05730-w

**Published:** 2022-02-01

**Authors:** Oksana A. Lenina, Konstantin A. Petrov

**Affiliations:** 1 Arbuzov Institute of Organic and Physical Chemistry, FRC Kazan Scientific Center of RAS, Arbuzov str., 8, Kazan, Russia 420088; 2 Kazan Federal University, 18 Kremlyovskaya str, Kazan, Russia 420008

**Keywords:** Drug discovery, Neuroscience, Physiology

## Abstract

Organophosphorus (OP) compounds that inhibit acetylcholinesterase are a common cause of poisoning worldwide, resulting in several hundred thousand deaths each year. The pathways activated during OP compound poisoning via overstimulation of muscarinic acetylcholine receptors (mAChRs) play a decisive role in toxidrome. The antidotal therapy includes atropine, which is a nonspecific blocker of all mAChR subtypes. Atropine is efficient for mitigating depression in respiratory control centers but does not benefit patients with OP-induced skeletal muscle weakness. By using an ex vivo model of OP-induced muscle weakness, we studied the effects of the M1/M4 mAChR antagonist pirenzepine and the M2/M4 mAChR antagonist methoctramine on the force of mouse diaphragm muscle contraction. It was shown that weakness caused by the application of paraoxon can be significantly prevented by methoctramine (1 µM). However, neither pirenzepine (0.1 µM) nor atropine (1 µM) was able to prevent muscle weakness. Moreover, the application of pirenzepine significantly reduced the positive effect of methoctramine. Thus, balanced modulation of neuromuscular synaptic transmission via M1 and M2 mAChRs contributes to paraoxon-induced muscle weakness. It was shown that methoctramine (10 µmol/kg, i.p.) and atropine (50 µmol/kg, i.p.) were equieffective toward increasing the survival of mice poisoned with a 2xLD_50_ dose of paraoxon.

## Introduction

Poisoning with organophosphorus (OP) compounds as pesticides is a serious public health problem, with over 200,000 deaths and several million nonfatal cases occurring every year^1^. Moreover, weaponized OP compounds (e.g., VX, sarin, Novichok) also represent an immense danger, as they could be readily accessible by terrorist organizations and can be used in criminal acts^2–5^.

OP compounds act as irreversible inhibitors of the enzymes acetylcholinesterase (AChE) and butyrylcholinesterase leading to the excessive accumulation of acetylcholine (ACh) in synapses and the subsequent overstimulation of cholinergic receptors^6^. This leads to so-called cholinergic syndrome. Death generally results from respiratory arrest due to a combination of peripheral acute cholinergic effects and central apnea^1^.

Emergency therapy for OP poisoning consists of atropine to prevent overstimulation of muscarinic acetylcholine receptors (mAChRs), oximes to reactivate the activity of cholinesterases and benzodiazepines to control seizures caused by weaponized OP compounds^7^.

It was shown that early rapid atropinization is efficient for mitigating autonomic signs and can prevent depression of respiratory control centers during poisoning with OP compounds^1^. However, it was shown that mAChRs have also been found at neuromuscular junctions (NMJs) and modulate ACh release. At vertebrate NMJs, activation of the M2 subtype of mAChRs inhibits but activation of the M1 subtype stimulates ACh release from motor nerve endings^8^. Downregulation of ACh release via activation of nicotinic acetylcholine receptors (nAChRs) is one of the pathways that decreases the safety factor of neuromuscular synaptic transmission during AChE inhibition^9,10^. Thus, it can be assumed that balanced regulation of neuromuscular synaptic transmission via activation of mAChRs can also contribute to OP-induced muscle weakness. The present study was designed to test this hypothesis using contractions of isolated mouse diaphragms as an ex vivo model of OP-induced muscle weakness. Paraoxon (POX) was used as a model OP compound. POX is the active metabolite of the agricultural insecticide parathion, which is converted to POX by the hepatic microsomal system^11^. The results presented here demonstrate that muscle weakness caused by ex vivo application of POX can be significantly prevented by pretreatment with the M2/M4 mAChR antagonist methoctramine. However, the M1/M4 mAChR antagonist pirenzepine or the nonspecific (M1-M5) mAChR antagonist atropine were unable to significantly prevent POX-induced impairment of mouse diaphragm contractility. In addition, the application of the M1/M4 mAChR blocker pirenzepine or the M1-M5 mAChR blocker atropine led to a significant decrease in the strength of muscle contractions, even when AChE was active. The application of pirenzepine after methoctramine led to a decrease in the protective effect of methoctramine against POX. Thus, modulation of synaptic transmission at NMJs via competition between pathways activated by M1 and M2 mAChRs contributes to POX-induced impairment of mouse diaphragm contractility. It was also shown that methoctramine at a dose of 8 mg/kg (10 µmol/kg) and atropine at a dose of 15 mg/kg (50 µmol/kg) were equieffective toward increasing the survival of mice poisoned with a 2xLD_50_ dose of POX (0.42 mg/kg).

## Results

### Effect of treatment with atropine, methoctramine or pirenzepine on paraoxon-induced muscle weakness ex vivo

**Figure 1:**
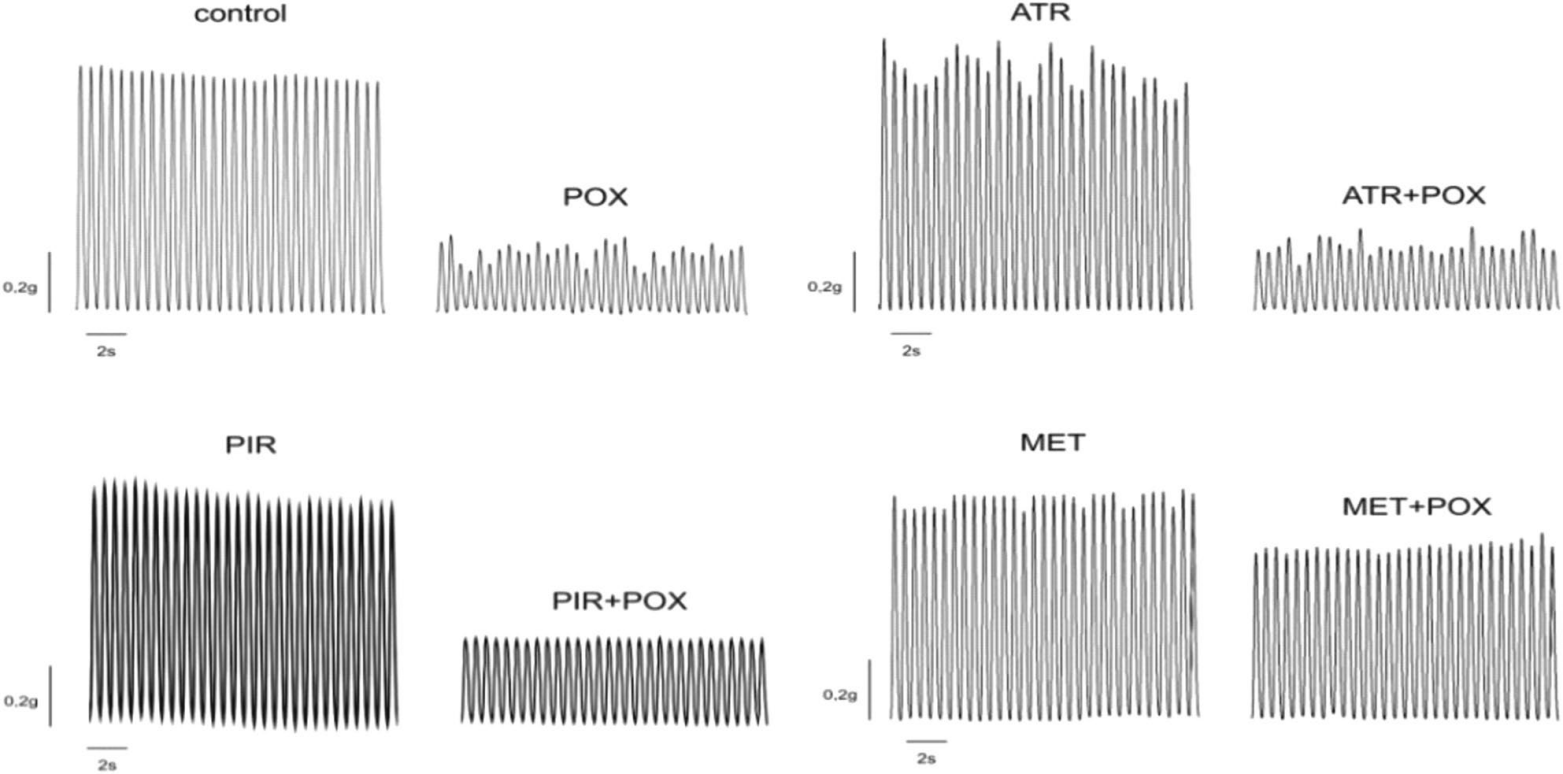
Representative contractions of mouse diaphragm muscle recorded in the presence of 0.5 μM paraoxon (POX), 1 µM atropine (ATR), 1 µM methoctramine (MET) and 0.1 µM pirenzepine (PIR).

**Figure 2:**
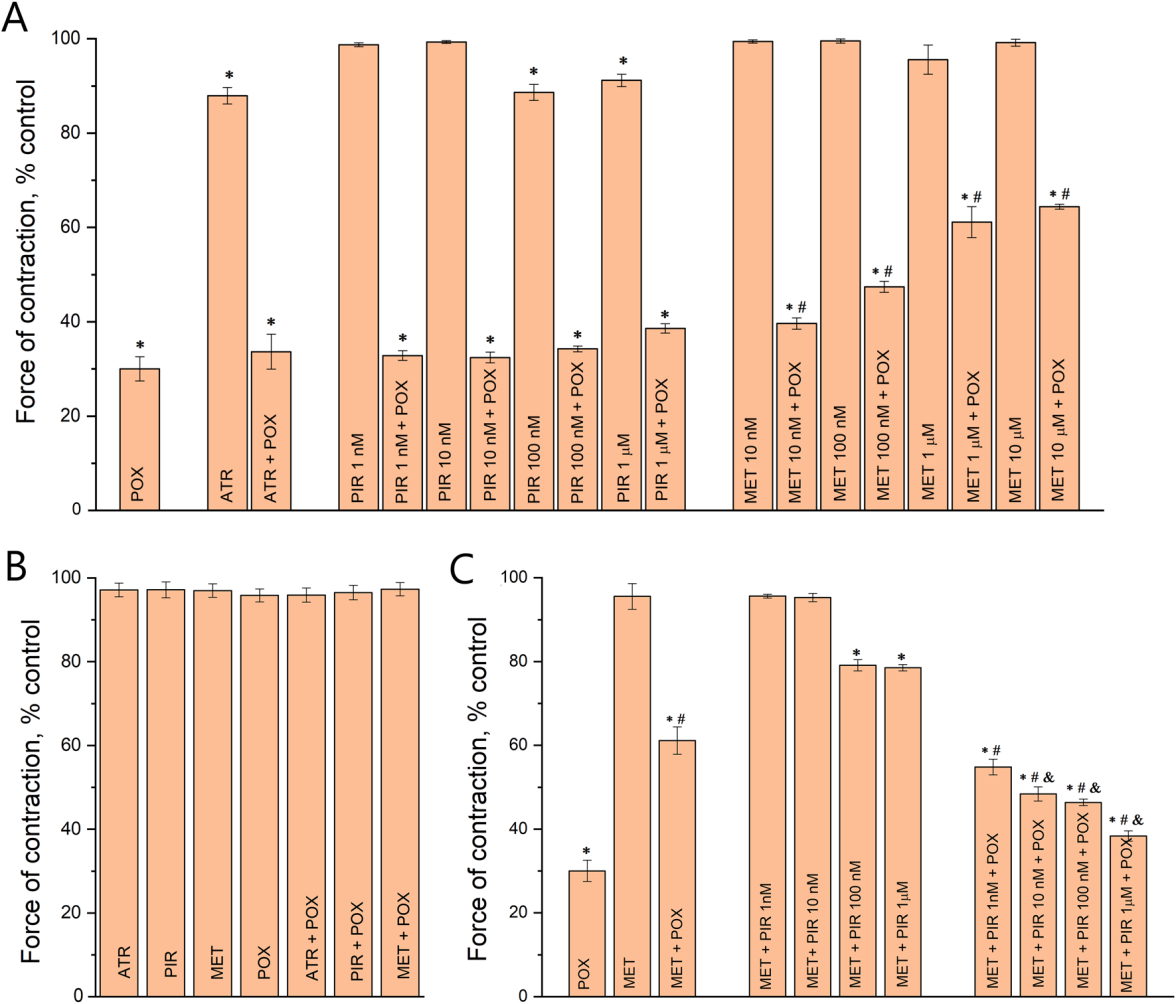
Relative changes in the force of diaphragm muscle contraction: (**A**) The mean force of diaphragm muscle contraction in presence of 0.5 μM paraoxon (POX), 1 µM atropine (ATR), 10 nM–10 µM methoctramine (MET) and 1 nM–1 µM pirenzepine (PIR). (**B**) Direct stimulation of diaphragm muscle in presence of 0.5 μM paraoxon (POX), 1 µM atropine (ATR), 1 µM methoctramine (MET) and 100 nM pirenzepine (PIR), when synaptic transmission was blocked by D-tubocurarine (1 µM). (**C**) Relative changes in the force of diaphragm muscle contraction in the presence of 1 µM methoctramine (MET) and after the subsequent application of pirenzepine (PIR) in concentrations of 1 nM–1 µM and 0.5 μM paraoxon (POX). The amplitude of muscle contractions in the control was taken as 100%. Data are expressed as the mean ± SEM. *p < 0.05 compared to contractions of intact control muscles. ^#^*p* < 0.05 compared to contractions in the presence of paraoxon. &*p* < 0.05 compared to contractions in the presence of methoctramine during nerve stimulation. Statistical analysis was performed using the Mann–Whitney test.

It was shown that incubating the mouse diaphragm for 30 min with 0.5 μM POX decreased the force of muscle contraction to 30 ± 1% of the control value (*p* = 0.0001; n = 10 muscles; Figs. 1, 2A). Preinhibition of the mouse diaphragm with atropine (1 µM) slightly but significantly decreased the diaphragm muscle contraction force to 88 ± 2% of the control value (*p* = 0.0001; n = 10 muscles). Pretreatment of the mouse diaphragm with pirenzepine in concentrations of 1 nM and 10 nM did not have a significant effect on the force of muscle contractions. However, when the concentration of pirenzepine was increased to 100 nM and 1 μM, the mean force of contractions significantly decreased to 89 ± 2% (*p* = 0.001; n = 10 muscles) and 91 ± 1% (*p* = 0.001; n = 10 muscles) of the control value, respectively (Figs. 1, 2A). Thus, blockade of M1 receptors ex vivo significantly decreased the mean force of diaphragm muscle contraction even when AChE was active. Importantly, methoctramine in concentrations of 10 nM, 100 nM, 1 µM or 10 µM did not have a significant effect on the force of contractions (Figs. 1, 2A).

Then, POX was applied after pretreatment with mAChR blockers (atropine, pirenzepine or methoctramine). It was shown that after pretreatment with atropine (1 µM), POX decreased the mean force of the diaphragm muscle to 34 ± 2% of the control (*p* = 0.0001; n = 10 muscles). A similar effect was observed after pretreatment with pirenzepine (1 nM, 10 nM, 100 nM, 1 µM), POX decreased the mean force of the diaphragm muscle to 33 ± 1%, 32 ± 1%, 34 ± 1% and 38 ± 1% of the control (*p* = 0.0001; n = 10 muscles), respectively (Figs. 1, 2A). However, after pretreatment with methoctramine, POX had a significantly smaller effect. The muscle contraction force was reduced only to 40 ± 1%, 47 ± 1%, 61 ± 3% and 64 ± 1% of the control value (*p* = 0.0001; n = 10 muscles) when methoctramine was applied in concentrations of 10 nM, 100 nM, 1 µM and 10 µM, respectively (Figs. 1, 2A).

In the next series of experiments, we blocked synaptic transmission at the NMJs with D-tubocurarine (1 µM) and then performed direct stimulation of muscle fibers by depolarizing impulses. It was shown that if muscle action potentials were triggered directly (via electrodes on muscle fibers), atropine (1 µM), methoctramine (1 µM), pirenzepine (100 nM) and POX (0.5 µM) had no significant effect on mouse diaphragm muscle contraction (Fig. 2B). This indicates that POX and mAChR blockers affect precisely synaptic transmission. Thus, ex vivo pretreatment of mouse diaphragm muscles with M2/M4 mAChR blocker but not with M1/M4 blocker or nonspecific M1-M5 blocker is able to decrease the action of POX on neuromuscular synaptic transmission.

It can be assumed that there is competition between M1 and M2 mAChR blockers under these experimental conditions. To test this hypothesis, pirenzepine was applied after methoctramine.

It was shown that after pretreatment with methoctramine (1 µM), pirenzepine in concentrations of 1 nM and 10 nM did not have a significant effect on the force of muscle contractions (Fig. 2C). However, in concentrations of 100 nM and 1 µM pirenzepine significantly decreased the mean force of diaphragm muscle contraction to 79 ± 2% (*p* = 0.003; n = 10 muscles) and to 79 ± 2% (*p* = 0.001; n = 10 muscles) of the control, respectively (Fig. 2C). Subsequent application of POX (0.5 μM), when pirenzepine was applied in concentrations of 1 nM, 10 nM, 100 nM and 1 µM, additionally reduced the contractions to 55 ± 2% (*p* = 0.001; n = 10 muscles), to 48 ± 2% (*p* = 0.01; n = 10 muscles), to 46 ± 1% (*p* = 0.001; n = 10 muscles) and to 38 ± 1% (*p* = 0.001; n = 10 muscles), respectively (Fig. 2C). Thus, blockade of M1 mAChRs in a concentration dependent manner reduces the positive effect of POX-induced muscle weakness treatment with M2 mAChR blocker.

### Effect of atropine or methoctramine treatment on paraoxon toxicity in vivo

During the next sets of experiments, we compared the efficiency of atropine and methoctramine as antidotes against poisoning with a 2xLD_50_ dose of POX (0.42 mg/kg). Atropine or methoctramine was administered intraperitoneally (i.p.) at different doses one minute after challenging the mice with POX. Atropine at a dose of 15 mg/kg had the strongest antidotal effect against POX (Table 1). Unexpectedly, methoctramine had the strongest antidotal effect against POX at a dose of 8 mg/kg (Table 2).

**Table 1:** Selection of atropine dose for antidotal therapy of mice poisoned by 2xLD_50_ of POX.

Group	n/N*
POX 0.42 mg/kg, i.p.	0/24
POX + Atropine 6 mg/kg, i.p.	0/24
POX + Atropine 8 mg/kg, i.p.	2/24
POX + Atropine 10 mg/kg, i.p.	6/24
POX + Atropine 15 mg/kg, i.p.	11/24
POX + Atropine 20 mg/kg, i.p.	10/24

*n number of mice survival 120 h after POX poisoning.

*N* total number of mice in the group. POX—0.42 mg/kg, i.p.

**Table 2:** Selection of methoctramine dose for antidotal therapy of mice poisoned by 2xLD_50_ of POX.

Group	n/N*
POX 0.42 mg/kg, i.p.	0/24
POX + methoctramine 3 mg/kg, i.p.	2/24
POX + methoctramine 4 mg/kg, i.p.	4/24
POX + methoctramine 6 mg/kg, i.p.	7/24
POX + methoctramine 8 mg/kg, i.p.	10/24
POX + methoctramine 10 mg/kg, i.p.	8/24

*n number of mice survival 120 h after POX poisoning.

*N* total number of mice in the group. POX—0.42 mg/kg, i.p.

**Figure 3:**
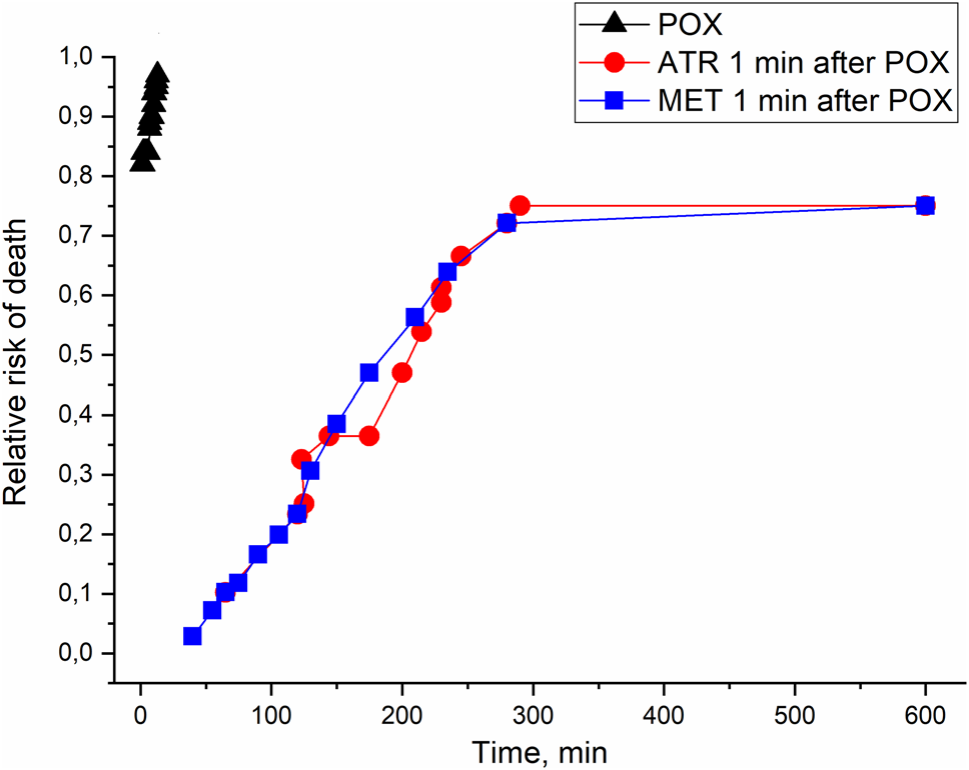
Cox analysis of survival data for mice treated with atropine (ATR) at a dose of 10 mg/kg or methoctramine (MET) at a dose of 8 mg/kg against a 2xLD50 dose (0.42 mg/kg, i.p.) of paraoxon (POX).

The relative risk (RR) of death after poisoning with POX as a function of precocity (from min to hours) of treatment with atropine at a dose of 15 mg/kg or methoctramine at a dose of 8 mg/kg was calculated according to Cox survival analysis over a period of 10 h^12^. As expected, it was shown that RR = 1 in mice exposed to a 2xLD_50_ dose of POX. However, among animals treated with 15 mg/kg atropine, mortality was lower (RR = 0.75; Fig. 3). The same RR of death after poisoning with POX was calculated, and mice were treated with methoctramine at a dose of 8 mg/kg. We compared the RR of death after antidotal therapy with atropine and methoctramine. There were no statistically significant differences between the efficacy of antidotal therapy with atropine at a dose of 15 mg/kg and methoctramine at a dose of 8 mg/kg (*p* = 0.45; n = 24 mice). Thus, the blockade of M2/M4 mAChRs is able to increase the survival of animals poisoned with POX as a blockade of M1–M5 mAChRs.

As a next step, we tried to replace atropine with methoctramine in the “cocktail” used for the treatment of OP poisoning. The results showed that administration of a "cocktail" of pralidoxime (30 mg/kg, i.p.), atropine (15 mg/kg, i.p.), diazepam (2 mg/kg, i.p.) one minute after poisoning saved 4 of 24 mice that received POX at a dose of 3xLD_50_ (0.63 mg/kg, i.p.) (Table 3). After replacing atropine with 8 mg/kg methoctramine, 5 of 24 mice that received POX at a dose of 3xLD_50_ survived (Table 3). Thus, methoctramine at a dose of 8 mg/kg has a similar effect to atropine at a dose of 15 mg/kg in the composition of the “cocktail”. However, it is important to note that the molecular weight of methoctramine is higher than that of atropine. Therefore, to compare the effective doses of methoctramine and atropine, we expressed them in terms of "μmol/kg". In this case, the effective dose of methoctramine (10 μmol/kg) was five times lower than the effective dose of atropine (50 μmol/kg). Thus, the results of this toxicological experiment suggest that blockade of M2/M4 mAChRs could be more effective for survival than nonselective blockade of all mAChR subtypes.

**Table 3:** Replacement of atropine on methoctramine in the “cocktail” used for the treatment of mice poisoned by 3xLD_50_ of POX.

Group	n/N*
POX + pralidoxime + atropine + diazepam	4/24
POX + pralidoxime + methactromine + diazepam	5/24

*n number of mice survival 120 h after POX poisoning.

*N* total number of mice in the group. POX—0.63 mg/kg, i.p.

Composition of the “cocktail”: pralidoxime (30 mg/kg, i.p.), atropine (15 mg/kg, i.p.) or methoctramine (8 mg/kg, i.p), diazepam (2 mg/kg, i.p.). Components of “cocktail” were i.p. administrated within 1 min after POX.

## Discussion

Atropine and oximes were first used for the treatment of OP compound poisoning in the 1950s, and this approach has not significantly changed over the last 60 years. However, a better understanding of the multiple respiratory complications of OP poisoning offers additional therapeutic opportunities^1^. Respiratory failure due to OP poisoning occurs with two distinctive clinical patterns. An early form is likely to be central and can be mitigated with rapid titration of antimuscarinic agents such as atropine. This is in contrast to late respiratory failure, which has been shown to be associated with neuromuscular dysfunction^13^. Unfortunately, standard antidotes have no benefit to patients who develop neuromuscular dysfunction. The only therapy currently available for late respiratory failure is intubation and mechanical ventilation, which is often required for a few weeks^14,15^.

One of the main objectives of the present study was to test the hypothesis that suboptimal inhibition of mAChRs at NMJs can decrease the efficiency of therapy for respiratory muscle weakness following OP poisoning.

mAChRs are members of the G-protein-coupled receptor family and consist of five distinct subtypes of mAChRs, denoted M1, M2, M3, M4, and M5. M1, M3 and M5 mAChRs couple to the G_q/11_ proteins to activate phospholipase C, whereas M2 and M4 couple to the G_i/o_ proteins, thereby stimulating downstream activities, such as inhibition of adenylyl cyclase^16^. The degree of interplay of these pathways in the central and peripheral nervous systems during AChE inhibition remains to be revealed.

It has been found that mAChRs of at least four subtypes (M1, M2, M3 and M4) are present at the NMJs^17^. It is known that at NMJs and mAChRs participate in the autoregulation of ACh release^10^. In some cases, exogenous mAChR agonists can suppress ACh release, while in other situations, they conversely enhance ACh release^18–22^. It was shown that the activation of M2 mAChRs is responsible for the depressive effect and that the activation of M1 mAChRs stimulates the process of ACh release^23–26^. A very similar effect was described in central synapses, and blockade of M2 mAChRs led to an increase in ACh release^27^.

The dominant mechanism of ACh-induced muscle paralysis is the depolarization block of muscle action potential generation, which is similar to the action of depolarizing myorelaxants (e.g., succinylcholine)^28,29^. Thus, the usefulness of antidepolarizing drugs targeting postsynaptic nAChRs is mainly discussed mainly as a potential therapy for OP-induced respiratory muscle weakness^14^. However, because the main parameter changing during the process of autoregulation of ACh release is the amplitude of the excitatory postsynaptic potentials, such balanced regulation via activation of M1 and M2 mAChRs can also be related to the modulation of the safety factor of synaptic transmission during OP poisoning.

Unfortunately, the level of ACh release cannot be directly estimated after inhibition of AChE at the NMJs due to nonlinear summation of the postsynaptic effect of the individual ACh quanta^30^. Nevertheless, taking the present data on muscle contraction and the abovementioned studies together, it can be suggested that presynaptic M2 mAChRs involved in the downregulation of ACh release at NMJs can be considered a new target for the treatment of OP-induced respiratory muscle weakness. In addition, the effects of M1 mAChR blockade that decrease the efficiency of the treatment could explain the low efficacy of atropine in the therapy of OP-induced late respiratory failure.

The mechanisms of early respiratory failure following acute OP poisoning are also not fully understood. Animal studies support the idea that early OP-induced respiratory failure results from the effects of OP compounds on muscarinic brainstem circuits, thereby interfering with respiratory rhythmogenesis and local pulmonary muscarinic effects (e.g., bronchoconstriction and bronchorrhea)^13,31–34^. The results of our toxicological experiments raise the question about the possible important role of M2 and/or M4 mAChRs during the acute phase of OP poisoning because antidotal treatment with methoctramine has the same effect as atropine. However, the possibility of cross-talk between mAChRs of different types could also explain the results of these toxicological experiments. The cross-talk between M1/M2 and M2/M3 mAChRs has been described^35,36^. In these complexes of mAChRs, one pathway can modulate the response of the second pathway^37^.

The above findings support the hypothesis that specific blockers for selected subtypes of mAChRs could be efficient for the treatment of OP poisoning. Thus, further studies are needed to determine the contributions of the different mAChR pathways activated during OP poisoning.

## Methods

### Ex vivo twitch tension measurements

All experiments involving animals were performed in accordance with the guidelines set forth by the European Union Council Directive 2010/63/EU, and conducted in accordance with ARRIVE guidelines, the protocol of experiments approved by the Animal Care and Use Committee of Kazan Federal University. CD-1 mice weighing 25–30 g, 6-week old, were purchased from the Laboratory Animal Breeding Facility (Branch of Shemyakin-Ovchinnikov Institute of Bioorganic Chemistry, Puschino, Moscow Region, Russia) and were allowed to acclimate to their environment in vivarium for at least 1 week before experiments. Animals were kept in sawdust-lined plastic cages in a well-ventilated room at 20–22 °C in a 12-h light/dark cycle, 60–70% relative humidity and given ad libitum access to food and water.

Ex vivo twitch tension measurements was performed as previously described^38^. Hemidiaphragm muscles with their associated phrenic nerves were bathed in oxygenated Ringer-Krebs’ solution at 25 °C. For twitch tension measurements the force sensor TRI201AD (AD Instruments, Sydney, Australia) was used. Contractions were evoked by stimulating the phrenic nerve via wire electrodes by supramaximal current pulses, 0.1 ms in duration. Data were recorded using Power Lab system and LabChart 6 software (AD Instruments, Sydney, Australia, https://www.adinstruments.com/products/labchart). Paraoxon (Sigma-Aldrich, St. Louis, MO, USA), atropine (Sigma-Aldrich, St. Louis, MO, USA) pirenzepine (Sigma-Aldrich, St. Louis, MO, USA) and methoctromine (Sigma-Aldrich, St. Louis, MO, USA) were applied in Ringer-Krebs solution.

Data were expressed as mean ± SEM. Drug effect was expressed as percentage of contraction amplitude in control. Statistical significance was assessed by Mann–Whitney test at the level of *p* < 0.05.

### In vivo antidotal therapy of poisoning with paraoxon

Animals were observed for 120 h after i.p. injection of POX. POX LD_50_, dose (in mg/kg) causing lethal effects in 50% of CD-1 mice was determined previously^38^. Atropine or methoctramine were i.p. administered at different doses 1 min after i.p. injection of 2xLD_50_ of POX (0.42 mg/kg). The ratio of number of mice surviving after intoxication with POX to the total number of mice in each group was used as a criterion of efficiency of antidotal therapy.

COX analysis of the RR of death was performed using SPSS Statistics software (IBM, USA). Statistical significance was assessed by Mann–Whitney U test at the level of *p* < 0.05.

## Abbreviations

ACh: Acetylcholine
AChE: Acetylcholinesterase
CNS: Central nervous system
i.p.: Intraperitoneal
LD50: Median lethal dose
mAChRs: Muscarinic acetylcholine receptors
nAChRs: Nicotinic acetylcholine receptors
NMJ: Neuromuscular junction
OP: Organophosphorus
POX: Paraoxon
